# Schuylkill Valley Veterinary Medical Association

**Published:** 1895-01

**Authors:** J. W. Sallade

**Affiliations:** Corresponding Secretary


Secretary.

Fort Leavenworth, Kansas, December 19, 1894.

### SCHUYLKILL VALLEY VETERINARY MEDICAL ASSOCIATION.

The fourth quarterly meeting of the Schuylkill Valley Veterinary Medical Association was held in the rooms of the Board of Health of the city of Reading, Wednesday, December 19, 1894. Dr. S. G. Burkholder, the President, called the meeting to order, and Dr. U. G. Fridirici, Recording Secretary, called the roll. The Corresponding Secretary of the Association, Dr. Jas. W. Sallade, of Pottsville, was present, and Dr. F. H. McCarthy, the Treasurer, as well as the Trustees, Drs. Otto Noack, of Reading; Eli Snyder, Orwigsburg; J. C. Faughnan, Shamokin, were also present.

This Association was organized April 29, 1893, and now includes members of Lebanon, Carbon, Lehigh, Montgomery, Luzerne, Northumberland, Schuylkill, and Lancaster counties, and promises to be a very active organization.

Dr. Noack, of Reading, read a paper on "Milk and Meat Inspection," giving the law and practice in his native country, Germany.

The meeting was further entertained by a paper on "Purpura Hemorrhagica,"[1] by Dr. J. W. Sallade, laying great stress upon better ventilation and watering for the better handling of such cases.

The meeting indorsed the proposed legislative act for the creation of a "Live-stock Sanitary Commission."

Dr. J. W. Sallade,
Corresponding Secretary.

[1] Printed in this number of this Journal.

